# How do Polish pharmacy staff evaluate own qualifications, competences, relevance, motivation, effectiveness in health promotion?

**DOI:** 10.1093/heapro/daab043

**Published:** 2021-06-24

**Authors:** Dorota Raczkiewicz, Beata Sarecka-Hujar, Adrianna Pawełczak-Barszczowska, Iwona Bojar

**Affiliations:** Department of Medical Statistics, School of Public Health, Center of Postgraduate Medical Education, Kleczewska str 61/63, 01-826 Warsaw, Poland; Department of Basic Biomedical Science, Faculty of Pharmaceutical Sciences in Sosnowiec, Medical University of Silesia in Katowice, Kasztanowa str 3, 41-200 Sosnowiec, Poland; Zentiva, Bonifraterska str 17, 00-203 Warszawa, Poland; Department of Women’s Health, Institute of Rural Health, Jaczewskiego str 2, 20-090, Lublin, Poland

**Keywords:** pharmacists, pharmacy staff, pharmacies, health promotion, health education

## Abstract

Health promotion programmes are complex and need to engage all health care specialists, including pharmacists. Pharmacies are considered as a potentially ideal place for health promotion and education. To evaluate own qualifications, competences, relevance, motivation and effectiveness of Polish pharmacy staff with regard to health-related information provided to patients, which contributes to health promotion. 308 pharmacy staff from Lublin (Poland) were surveyed with a questionnaire prepared by the researchers and piloted previously. It consisted of 5 domains: qualifications, competences, relevance, motivation, and effectiveness of health promotion. Items in each domain were scored by respondents in 1 − 10 scale where 1 is ‘very low’ and 10 is ‘very high’. Pharmacy staff rated the relevance of health promotion the highest, while the lowest—own competences and effectiveness in health promotion. Female pharmacy staff assessed the relevance of health promotion significantly higher than males (7.1 vs. 6.1, *p* = 0.005). Higher self-assessments of qualifications, competences, relevance, motivation and effectiveness in health promotion were provided by: the youngest pharmacy staff, those with a short period of employment, and pharmacy staff working in pharmacies employing up to 3 persons. Qualification and competences in health promotion were assessed higher by pharmacy technicians and masters of pharmacy with post-graduate studies or professional specialization, or Ph.D. than by masters of pharmacy. Relevance and effectiveness in health promotion were evaluated higher by pharmacy staff in pharmacies serving more than 100 customers daily. There is a need to improve qualifications, competences, relevance, motivation and effectiveness in health promotion conducted by Polish pharmacy staff.

## INTRODUCTION

A highly healthy society is one of the priorities of the European Union (Treaty on European Union, Maastricht 1992). Health promotion as well as disease prevention, that is programmes increasing men's interest in their health, are used to achieve this goal (Kumar and Preetha, 2012).

Activities in the area of public health are comprehensive and need the involvement of healthcare professionals, also including pharmacists, whose role in health promotion and disease-prevention is dynamically changing (Laliberté *et al.*, 2012; DiPietro Mager and Farris, 2016). In many countries, pharmacists play an important role in health promotion (Ecarnot *et al.*, 2019; Resolution the Committee of Ministers, 2020). This is especially visible in Anglo-Saxon countries where pharmacists are engaged in vaccine implementation (Ecarnot *et al.*, 2019). In Italy, pharmacies participate in the health education programs and prevention campaigns. In addition, the resolution of the Italian Health Department indicated the role of community pharmacists in promoting vaccinations (Scarpitta *et al.*, 2019). To use in full the potential of pharmacies to deliver health benefits to the population all health professionals should collaborate with pharmacists (Resolution the Committee of Ministers, 2020). The Pharmaceutical Group of the European Union (PGEU) Vision for Community Pharmacy 2030 indicates that local pharmacists should be supported in offering health screening, as well as health promotion and education to help reduce the overall burden of chronic diseases (The PGEU Vision for Community Pharmacy, 2030). Such an engagement of pharmacists in preventive campaigns and health promotion allows to improve adherence to therapeutic recommendations, may also reduce possible side effects and drug interactions as well as may have an economic dimension, since it reduces the costs of potential treatment (Houle *et al.*, 2012; Hendrie *et al.*, 2014; Simpson *et al.*, 2015; Isetts *et al.*, 2016). The Polish Act on Pharmaceutical Chambers indicates that the role of pharmacists is to protect public health and in cooperation with patients and doctors, to supervise the proper course of pharmacotherapy in order to obtain specific effects improving patients' quality of life (Act of Laws on pharmacy chambers, 2008).

The literature data indicated the legitimacy of involving pharmacists in a wide range of patient care, including disease prevention, health education, participation in an immunization and vaccination process, and a support of addiction treatment (Blanchet, 2015; Um *et al.*, 2015; Dirks-Naylor *et al.*, 2018; Morillo-Verdugo *et al.*, 2018). When pharmacists in Edmonton (Alberta, Canada) were incorporated into primary care tasks, it resulted in a significant improvement in blood pressure control which decreased the risk of heart and cardiovascular disease in patients with type 2 diabetes, and in turn in a significant reduction of the cost of potential treatment (Simpson *et al.*, 2015).

Studies from different countries demonstrated among others the possible implementation of a weight control program in overweight and obese patients in Australian pharmacies (Um *et al.*, 2015), and the novel model for blood pressure control screening and counselling in community pharmacies in Poznań (Poland) (Waszyk-Nowaczyk *et al.*, 2020).

In turn, an intensive pharmaceutical care using individual motivational interview and periodic contact by text messages about health promotion helped to lower the Framingham-score from high/very high to moderate/low cardiovascular risk in almost 21% of Spanish patients with HIV and almost 38% of them stopped smoking (Morillo-Verdugo *et al.*, 2018). The study by Watkins *et al.* (2020) indicated that community pharmacists can have a positive impact on reduction of number of medications per patient as well as systolic and diastolic blood pressures in patients with multiple chronic conditions through health coaching services.

Among the main obstacles in proper health promotion, pharmacists indicate being overloaded with work, not having enough time for a conversation with a patient, and not having enough skills and practice when dealing with a patient. The Lack of legal regulations, the lack of an appropriate place for a conversation with a patient, financial predictors and sometimes the patient's disregard for communication are also reported as barriers in efficient health education and disease prevention (Muijrers *et al.*, 2003; Arora *et al.*, 2015; Kazaryan and Sevikyan, 2015; Rayes *et al.*, 2015; Hyoguchi *et al.*, 2016; Devraj and Young, 2017; Gregório *et al.*, 2017).

This study aimed to evaluate own qualifications, competences, relevance, motivation and effectiveness of pharmacy staff in Poland with regard to health-related information provided to their patients, which contributes to health promotion.

## METHODS

### Study group

This study is the second part of a research project devoted to health promotion and health education by pharmacy staff in community pharmacies in Poland. The first part of the project was aimed to evaluate the readiness of Polish pharmacy staff to implement health promotion and educational activities, including: systemic solutions for health promotion, their readiness as a professional group and their personal readiness to promote health (Bojar *et al.*, 2020).

The survey was carried out in 2017–2018 in a sample of pharmacy staff (pharmacists and pharmacy technicians) employed in community pharmacies in the Lublin region, Poland. Community pharmacies in this region are representative of community pharmacies in the other 15 regions of Poland, as they all function in the same manner and are governed by the same legal regulations. In 2017, in the Lublin region there were 865 pharmacies listed on the National Health Fund (NHF) Lublin Region website. Due to some financial and organizational limitations, 140 pharmacies from this list were selected for the study, which constitutes 16% of all the pharmacies in the Lublin region. A systematic sample of pharmacies was selected, where every sixth pharmacy from the NHF list was chosen. Each selected pharmacy was sent 5 copies of the survey and asked to fill it in by the pharmacy staff. The total of 368 surveys were sent back to us. The response rate was 52%. A total number of 308 correctly completed questionnaires were included in the study.

### Research instrument

The research tool was a questionnaire prepared by the authors. It was piloted in a preliminary survey that was conducted in a sample of 20 pharmacy staff. Based on comments of respondents some of the items of questionnaire were modified for greater clarity. The survey questionnaire consisted of 2 parts. Part 1 of the questionnaire contained items concerning the examined pharmacy staff and the pharmacies where they worked. The respondents were asked about: gender, age, level of education, and job seniority. The pharmacies were analysed based on: number of employees, location, and the mean daily number of customers.

Part 2 of the questionnaire included 5 questions to pharmacy staff:

1. How do you assess your qualifications to promote health in a pharmacy in the following items?
2. How do you assess your formal competences to promote health in a pharmacy in the following items?
3. How do you assess relevance of health promotion in a pharmacy in the following items?
4. How do you assess your motivations to promote health in a pharmacy in the following items?
5. How do you assess your effectiveness in health promotion in a pharmacy in the following items?

Each question contained 36 items in 3 groups (subdomains): health knowledge (11 items); disease prevention (9 items); coping with health problems (16 items). In total, pharmacy staff evaluated180 items (5 questions × 36 items). All items were assessed by the respondents from 1 to 10; where 1 was ‘very low’, 10 was ‘very high’. The scale mid-point was 5.5; that is results significantly lower than 5.5 were considered negative, significantly higher than 5.5 were considered positive and not significantly different from 5.5 as neutral.

**Fig. 1:**
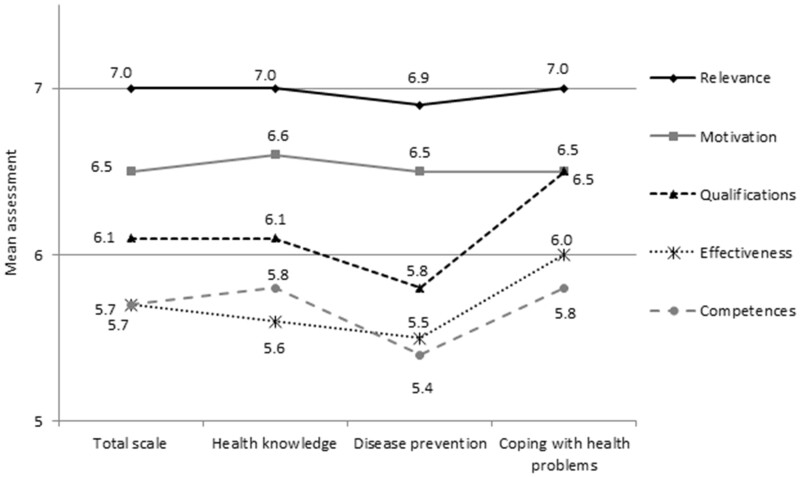
Qualifications, competences, relevance, motivation and effectiveness in health promotion—pharmacy staff’s self-assessments of domains and subdomains. *Notes:* Scale 1–10; where 1—very low, 10—very high.

**Fig. 2:**
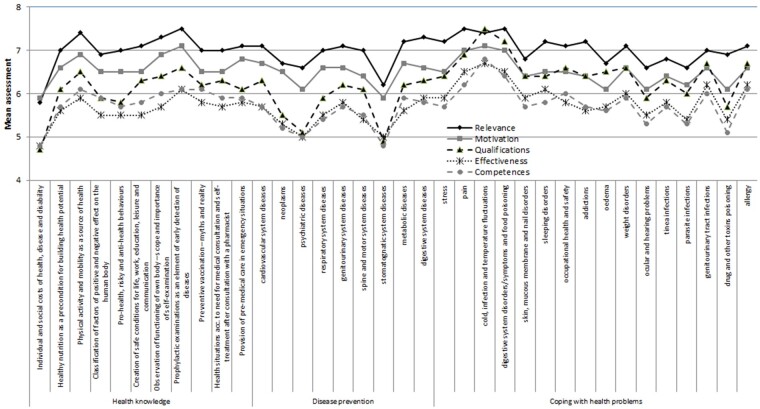
Qualifications, competences, relevance, motivation and effectiveness in health promotion—pharmacy staff’s self-assessments of items in domains and subdomains. *Notes:* Scale 1–10; where 1—very low, 10—very high.

We calculated the ratings of the 5 domains: qualifications (question 1), competences (question 2), relevance (question 3), motivations (question 4) and effectiveness (question 5) as a mean of 36 items in each domain. We also calculated the rating of the 3 groups (subdomains) of items in each domain: health knowledge (as a mean of 11 items); disease prevention (as a mean of 9 items); coping with health problems (as a mean of 16 items). All domains and subdomains are listed on Figure 1, while all items in domains and subdomains are listed on Figure 2.

Table 1 presents Cronbach’s α and average inter-correlation coefficient between items *r* for domains and subdomains of self-assessment of qualifications, competences, relevance, motivation and effectiveness in health promotion by pharmacy staff. The reliability of the 5 domains and the 3 subdomains in every 5 domains (total 15 subdomains) was investigated using Cronbach’s α and average inter-correlation coefficient between items *r*. Based on factor analysis, the division of items into 3 subdomains was confirmed, explaining 67.9% of total variance for the domain qualifications; 74.4% for the domain competences; 77.5% for the domain relevance; 79.7% for the domain motivation; and 80.1% for the domain effectiveness of health promotion. High values of Cronbach’s α, strong inter-correlation coefficients between items, and a high percentage of the explained variance indicated high reliability and internal consistency of each of 5 domains in general, and in the 3 subdomains, which allows them to be used in further analyses.

**Table 1:** Cronbach’s α and average inter-correlation coefficient between items *r* for domains and subdomains of self-assessment of qualifications, competences, relevance, motivation and effectiveness in health promotion by pharmacy staff

Domain	Domains in general		Subdomains					
			Health knowledge		Diseases prevention		Coping with health problems	
	α	*r*	α	*r*	α	*r*	α	*r*
Qualifications	0.976	0.547	0.929	0.554	0.943	0.659	0.968	0.661
Competences	0.986	0.669	0.967	0.732	0.963	0.756	0.977	0.733
Relevance	0.986	0.676	0.961	0.705	0.966	0.782	0.980	0.761
Motivation	0.991	0.753	0.973	0.775	0.977	0.835	0.983	0.786
Effectiveness	0.989	0.715	0.972	0.771	0.973	0.805	0.979	0.748

The significance level was assumed at 0.05.

### Statistical methods

The statistical analysis was conducted with STATISTICA 12 software (Statsoft, Poland). The absolute numbers (*n*) and percentages (%) were estimated for the categorical variables. Arithmetic mean (M) and standard deviation (SD) were estimated for continuous variables. The following statistical tests were used:

- one sample *t*-test against a value of 5.5 to check whether own qualifications, competences, relevance, motivation and effectiveness in health promotion are assessed neutrally or significantly positively or significantly negatively by pharmacy staff;
- two unpaired samples *t*-test to compare qualifications, competences, relevance, motivation and effectiveness in health promotion, between men and women;
- F-test analysis of variance to compare qualifications, competences, relevance, motivation and effectiveness in health promotion, between 3 levels of education, 4 periods of employment, 3 intervals of number of employees, 4 locations of pharmacies, 3 intervals of average number of customers served daily;
- Pearson’s correlation coefficient r to correlate qualifications, competences, relevance, motivation and effectiveness in health promotion, with pharmacy staff's age.

### Ethical approval

Surveys involving humans are not considered medical research in Poland and therefore do not require formal approval of the Local Ethics Committee. Obviously, the survey conducted in this study was in line with the ethical standards of the institutional bioethical commission and the Helsinki Declaration (1964). Although participation in the study was anonymous and voluntary, each surveyed participant gave verbal consent.

## RESULTS

### Characteristics of the surveyed pharmacy staff and characteristics of the pharmacies where they worked

Table 2 shows sample characteristics. The mean respondents’ age was 38 ± 11-years-old. In the study group, females and pharmacy staff with age range 30–39-years-old predominated (91% and 40%, respectively). The largest number of respondents had a university education (63%), and the period of their employment was from 5 to 14 years (44%). The largest number of pharmacies employed 4–5 persons (46%), they were located near health care institutions (59%), and served 100–200 customers a day on average. The same study group’s characteristics was presented in our previous research on readiness of Polish pharmacy staff to engage in health promotion and educational activities (Bojar *et al.*, 2020).

**Table 2:** Sample characteristics

Variable	Category	Estimate
Total sample	−	308 (100.00)
Gender, *n* (%)	Females	279 (90.58)
	Males	29 (9.42)
Age (years)	M±SD	38.0 ± 11.1
Age group (years)	20–29	73 (23.70)
	30–39	124 (40.26)
	40–49	50 (16.23)
	50–59	47 (15.26)
	60–65	14 (4.55)
Level of education, *n* (%)	Pharmacy technicians	60 (19.48)
	Masters of pharmacy	194 (62.99)
	Masters of pharmacy with post-graduate studies or professional specialization, or Ph.D.	54 (17.53)
Period of employment (years), *n* (%)	up to 5	72 (23.38)
	5 − 14	136 (44.16)
	15 − 25	48 (15.58)
	More than 25	52 (16.89)
No. of persons employed in a pharmacy, *n* (%)	Up to 3	50 (16.23)
	4 − 5	141 (45.78)
	More than 5	117 (37.99)
Location of pharmacies, *n* (%)	In the vicinity of a health care institution	182 (59.09)
	In an agglomeration with intensified pedestrian traffic	61 (19.81)
	In a residential area	29 (9.42)
	Others	36 (11.69)
Mean number of customers served by pharmacies daily, *n* (%)	Up to 100	97 (31.49)
	100 − 200	153 (49.68)
	More than 200	58 (18.83)

M, mean; SD, standard deviation.

### Self-assessment of pharmacy staff in domains and subdomains

The pharmacy staff evaluated the relevance of health promotion the highest (overall evaluation 7.0 ± 1.7), followed by their motivation for health promotion (6.5 ± 2.0) and qualifications for health promotion (6.1 ± 1.4) (Figure 1). The lowest evaluations were reported for their own effectiveness and competences for health promotion (5.7 ± 1.9 and 5.7 ± 1.7, respectively). Nearly all of the overall self-assessments were significantly positive (*p* < 0.001 for relevance, motivation and qualifications; *p* = 0.039 for competences), while an overall self-assessment of effectiveness was neutral (undecided) (*p* = 0.065).

Self-assessments of relevance were similar in 3 subdomains: health knowledge (7.0 ± 1.8), disease prevention (6.9 ± 2.0), and coping with health problems (7.0 ± 1.8). Also, self-assessments of motivation were similar in 3 subdomains: health knowledge (6.6 ± 2.0), disease prevention (6.5 ± 2.3), and coping with health problems (6.5 ± 2.1). In turn, self-assessments of qualification, effectiveness and competences differed between 3 subdomains. Qualifications in disease prevention were evaluated the lowest (5.8 ± 1.6), qualifications for coping with health problems were evaluated the highest (6.5 ± 1.5), and health knowledge was assessed between the two subdomains mentioned above (6.1 ± 1.6). The effectiveness of coping with health problems was evaluated significantly higher (6.0 ± 1.9) than the effectiveness of health knowledge and disease prevention (5.6 ± 1.9 and 5.5 ± 2.2, respectively). Competences in disease prevention obtained significantly lower evaluations (5.4 ± 2.0) than competences in health knowledge and coping with health problems (5.8 ± 1.9 and 5.8 ± 1.6, respectively).

### Items which obtained the highest and the lowest evaluations

The surveyed pharmacy staff provided the lowest evaluations concerning their qualifications, competences, relevance, motivation and effectiveness in the items as follows: individual and social costs of health, diseases and disabilities, psychiatric disease prevention, and disease prevention of the stomatognatic system (Figure 2).

The pharmacy staff assessed their qualifications, competences, relevance, motivation and effectiveness in the following items as the highest: physical activity and mobility as a source of health, prophylactic examinations as an element of early detection of diseases, prevention of cold, infection and temperature fluctuations.

### Correlations between self-assessments in domains and the surveyed pharmacy staff’s characteristics

Table 3 presents self-assessments of qualifications, competences, relevance, motivation and effectiveness in health promotion versus surveyed pharmacy staff’s characteristics. Female pharmacy staff assessed the relevance of health promotion significantly higher than male pharmacy staff (7.1 ± 1.8 vs. 6.1 ± 1.3, *p* = 0.005). Assessments of the remaining 4 domains did not significantly differ between males and females (Table 3).

**Table 3:** Self-assessments of qualifications, competences, relevance, motivation and effectiveness in health promotion versus surveyed pharmacy staff’s characteristics

Variable	Category, parameter	Qualifications	Competences	Relevance	Motivation	Effectiveness
Gender	Females, M±SD	6.1 ± 1.4	5.7 ± 1.8	7.1 ± 1.8	6.6 ± 2.1	5.7 ± 1.9
	Males, M±SD	6.2 ± 1.6	5.5 ± 1.6	6.1 ± 1.3	6.1 ± 1.8	5.7 ± 1.8
	*p* (t)	0.793	0.431	**0.005**	0.190	0.996
Age (years)	*r*	–0.217	–0.167	–0.288	–0.202	–0.214
	*p*	**<0.001**	**0.003**	**<0.001**	**<0.001**	**<0.001**
Age group (years)	20–29, M±SD	6.8 ± 1.4	6.5 ± 1.4	7.5 ± 1.6	7.6 ± 1.6	6.7 ± 1.7
	30–39, M±SD	6.1 ± 1.4	5.5 ± 1.8	7.3 ± 1.7	6.4 ± 2.3	5.5 ± 2.1
	40–49, M±SD	5.4 ± 1.3	5.1 ± 1.7	6.1 ± 1.8	5.6 ± 1.6	5.0 ± 1.7
	50–65, M±SD	5.9 ± 1.3	5.6 ± 1.7	6.3 ± 1.6	6.4 ± 1.7	5.4 ± 1.4
	*p* (F)	**<0.001**	**0.003**	**<0.001**	**<0.001**	**<0.001**
Level of education	Pharmacy technicians, M ± SD	6.3 ± 1.5	6.1 ± 1.7	7.0 ± 1.8	6.7 ± 2.0	6.0 ± 2.0
	Masters of pharmacy M ± SD	6.0 ± 1.4	5.5 ± 1.7	6.9 ± 1.6	6.4 ± 2.1	5.6 ± 2.0
	Masters of pharmacy with post-graduate studies or professional specialization, or Ph.D., M ± SD	6.6 ± 1.4	6.2 ± 1.8	7.2 ± 2.0	6.9 ± 1.8	5.6 ± 1.3
	*p* (F)	**0.025**	**0.004**	0.520	0.249	0.403
Period of employment (years)	Up to 5, M ± SD	6.7 ± 1.3	6.5 ± 1.6	7.5 ± 1.5	7.5 ± 1.6	6.7 ± 1.8
	5 − 14, M ± SD	6.0 ± 1.5	5.4 ± 1.7	7.2 ± 1.7	6.2 ± 2.3	5.3 ± 2.1
	15 − 25, M ± SD	5.9 ± 1.5	5.5 ± 1.8	6.4 ± 2.0	6.2 ± 1.9	5.3 ± 1.6
	More than 25, M ± SD	5.9 ± 1.1	5.6 ± 1.5	6.2 ± 1.5	6.4 ± 1.5	5.5 ± 1.2
	*p* (F)	**0.005**	**<0.001**	**<0.001**	**<0.001**	**<0.001**

Scale 1–10; where 1, very low; 10, very high. M, mean; SD, standard deviation; t, Student’s test for two unpaired samples, *F*, test of analysis of variance; *r*, Pearson’s correlation coefficient; *p* for significant differences or correlations is in bold.

Significant negative correlations were found between the respondents’ age and their self-assessments of qualifications, competences, relevance, motivation and effectiveness in health promotion (*r* < 0; *p* < 0.05). This means that the younger the pharmacy staff was, the higher evaluations they provided, on average, while older respondents provided lower evaluations of their qualification, competences, relevance, motivation and effectiveness in health promotion.

Self-assessments of qualification and competences for health promotion significantly depended on the level of education (*p* = 0.025 and *p* = 0.004, respectively). The respondents with university-only education assessed their qualifications for health promotion the lowest (6.0 ± 1.4), the respondents with more than university education (university and post-graduate study or professional specialization, or Ph.D.)—the highest (6.6 ± 1.4), and pharmacy technicians provided assessments between the two groups mentioned above (6.3 ± 1.5). The examined pharmacy staff with university-only education evaluated their competences for health promotion significantly lower (5.5 ± 1.7), compared to those with more than university education (6.2 ± 1.8) or pharmacy technicians (6.1 ± 1.7). In turn, self-assessments of relevance, motivation and effectiveness in health promotion did not significantly depend on the level of education (*p* > 0.05).

The examined pharmacy staff with the shortest period of employment (up to 5 years) evaluated all domains of health promotion significantly higher: their qualifications, competences, relevance motivation and effectiveness, compared to respondents with a longer period of employment (*p* < 0.05). These domains were assessed significantly higher by 20-years-olds, lower by 30-years-olds, the lowest by pharmacy staff aged 40 or more years old.

### Correlations between self-assessments in domains and characteristics of pharmacies employing the respondents

Table 4 presents self-assessments of qualifications, competences, relevance, motivation and effectiveness in health promotion versus characteristics of pharmacies where surveyed pharmacy staff worked. The respondents from pharmacies employing up to 3 persons evaluated their qualifications, competences, relevance and motivation for health promotion significantly higher, than the respondents from pharmacies employing more staff. The effectiveness in health promotion was evaluated the highest by the respondents from pharmacies employing up to 3 persons, while the staff from pharmacies employing 4–5 persons provided the lowest evaluations, and the staff from pharmacies employing more than 5 persons scored between the two groups mentioned above.

**Table 4:** Self-assessments of qualifications, competences, relevance, motivation and effectiveness in health promotion versus characteristics of pharmacies where surveyed pharmacy staff worked

Variable	Category, parameter	Qualifications	Competences	Relevance	Motivation	Effectiveness
Number of persons employed in a pharmacy	Up to 3, M ± SD	7.0 ± 1.3	6.8 ± 1.6	7.6 ± 1.5	7.3 ± 1.7	6.8 ± 1.5
	4 − 5, M ± SD	6.1 ± 1.5	5.5 ± 1.9	6.8 ± 1.8	6.3 ± 2.1	5.2 ± 1.8
	More than 5, M ± SD	5.9 ± 1.3	5.5 ± 1.4	7.0 ± 1.7	6.4 ± 2.0	5.8 ± 1.9
	*p* (F)	**<0.001**	**<0.001**	**0.014**	**0.018**	**<0.001**
Location of pharmacies	In the vicinity of a health care institution, M ± SD	6.1 ± 1.4	5.6 ± 1.6	6.9 ± 1.6	6.3 ± 1.8	5.4 ± 1.7
	In a part of an agglomeration with intensified pedestrian traffic, M ± SD	6.1 ± 1.6	5.6 ± 1.8	6.8 ± 1.9	6.4 ± 2.4	6.2 ± 2.3
	In a residential area, M ± SD	6.2 ± 1.2	5.3 ± 1.9	6.6 ± 1.6	6.3 ± 1.7	5.4 ± 1.6
	Others, M ± SD	6.6 ± 1.7	6.6 ± 1.9	8.1 ± 1.6	7.8 ± 2.3	6.5 ± 1.9
	*p* (F)	0.253	**0.014**	**0.001**	**0.001**	**0.001**
Mean number of customers served by pharmacies daily	Up to 100, M ± SD	6.0 ± 1.3	5.4 ± 1.7	6.6 ± 1.8	6.2 ± 2.2	5.3 ± 2.1
	100 − 200, M ± SD	6.2 ± 1.6	5.8 ± 1.9	7.0 ± 1.7	6.7 ± 1.9	5.8 ± 1.8
	More than 200, M ± SD	6.2 ± 1.4	6.0 ± 1.5	7.4 ± 1.6	6.7 ± 2.2	6.1 ± 1.9
	*p* (F)	0.608	0.163	**0.024**	0.102	**0.020**

Scale 1–10; where 1, very low; 10, very high. M, mean; SD, standard deviation; *t*, Student’s test for two unpaired samples; *F*, test of analysis of variance; *r*, Pearson’s correlation coefficient; *p* for significant differences is in bold.

The surveyed staff from pharmacies located near health care institutions from agglomerations with increased pedestrian traffic or in residential areas, provided significantly lower evaluations of their competences, relevance, motivation and effectiveness of health promotion than those from pharmacies located in other places (*p* < 0.05). In turn, self-assessments of qualifications for health promotion did not significantly depend on the location of the pharmacies where the respondents were employed (*p* = 0.253).

Self-assessments of qualifications, competences and motivation for health promotion did not significantly depend on the mean daily number of customers served by the pharmacies where the respondents were employed (*p* > 0.05). Relevance and effectiveness were evaluated significantly lower by the staff from pharmacies serving up to 100 customers daily, than by the staff from pharmacies serving more customers daily (*p* = 0.024 and *p* = 0.020, respectively).

## DISCUSSION

Our study concerned five domains of health promotion by pharmacy staff in community pharmacies based on the author's questionnaire. The study showed that the surveyed pharmacy staff rated the relevance of health promotion the highest, their motivation to promote health as well as their qualification for health promotion was rated lower, whereas their effectiveness and competence to promote health were graded the lowest.

The role of pharmaceutical practice in many countries, including Poland, is changing from a traditional one, focusing solely on preparing and dispensing medications to a role focused on patient care. Educational programmes aimed at improving patients' health awareness and their attitude to health matters in general are an important part of the preamble to the Perspectives of Pharmacy Development until 2030 (Skowron *et al.*, 2016). However, the implementation of the proposed pharmaceutical care assumptions in Poland encounters some difficulties related to old habits or bad work organization, among others. An interest in expanding the role of pharmacists in community pharmacies in health education and disease prevention began to increase at the end of the last century. Canadian studies have shown that few pharmacists routinely performed activities related to disease prevention at that time. However, over 90% of pharmacists participating in the survey noted that the inclusion of preventive issues in their daily practice is very important. The lack of time and the lack of skills to provide such advice was pointed out as a barrier (O’Loughlin *et al.*, 1999). One of the earliest studies in this area showed that pharmacists from London willingly provided advice to patients, paying special attention to patients' requests for assistance (Smith, 1992). It was, however, demonstrated that pharmacists were focused mainly on providing information on the medicine dispensed, and in many cases they did not have an opportunity to discuss other issues related to health promotion (Smith, 1992). On the other hand, pharmacists in Kuwait engage in advising patients on how to use prescribed medicines according to the recommendations; they indicate possible side effects which may occur during using certain pharmaceutical preparations but they are less involved in advising on other health-promoting behaviour (Awad and Abahussain, 2010). Many studies also indicated the willingness of the pharmacists to acquire knowledge on health promotion. A high percentage of pharmacists in Sudan (over 70%) declare their readiness to participate in public health and their willingness to learn how to help to modify patients' behaviour in order to ensure a more effective health education of the society (Mohamed *et al.*, 2013). The authors demonstrated that almost 90% of pharmacists provide information on a healthy diet to patients, whereas almost 80% of them educate patients on obesity and weight reduction.

In the study by Laliberté *et al.* (2012), 571 pharmacists completed the survey, and the majority of them agreed that they should be more involved in health promotion and prevention, in particular in smoking cessation (84.3%), screening for hypertension (81.8%), diabetes mellitus (76.0%) and dyslipidaemia (56.9%), as well as sexual health (61.7% to 89.1%). According to the surveyed pharmacists, preventive services for the above mentioned health problems were given a ‘few times per week’ or a ‘few times per month’, whereas the activities related to infectious diseases and immunization took place a ‘few times per month’ or a ‘few times per year’. Simultaneously, the authors point out that the main organizational obstacles to a comprehensive health education and disease prevention is time, bad cooperation with representatives of other medical professionals but also other pharmacy personnel, as well as resources and financial issues (Laliberté *et al.*, 2012).

Waszyk-Nowaczyk and Simon (2009) emphasizes that, although medicines whose safety have been known are usually approved for OTC prescription, pharmacists must assume that an average patient does not possess knowledge which would guarantee them to carry out their treatment effectively and safely. For this reason, the process of patient education is of great importance. Other important factors which can help change the pharmacist's professional role and develop this profession towards a clinical one are, among others, wide knowledge of drug information sources, and their competent usage, as well as effective communication with both patients and doctors (Waszyk-Nowaczyk and Simon, 2009). Nowadays, the self-treatment, that is making decisions concerning therapy without consultations with a physician, is a common phenomenon. The percentage of Poles buying OTC drugs is very high; the recent study demonstrated that almost all of the 600 Polish adults surveyed use this type of medicines. Moreover, they do not consult their choices with a pharmacist (Majchrowska *et al.*, 2019). Similarly, a high percentage of patients take OTC drugs in other countries, despite existing differences in healthcare systems, and sometimes also in the availability of OTC drugs (Kim *et al.*, 2018; Mahrous, 2018; Tesfamariam *et al.*, 2019). Data indicate that pharmacists can engage in primary and secondary prevention of diseases, for example cardiovascular disease, by supporting the use of appropriate dietary supplements. The study by Waltz *et al.* (2016) demonstrated that pharmacists in the State of Alberta (Canada) can effectively help patients by presenting the benefits and the possible risks of taking dietary supplements.

This study showed that females, young pharmacy staff, those with post-graduate studies, a Ph.D. or a professional specialization in pharmacy, and those with a short period of employment had a higher self-assessment of some of the domains analysed in the study: qualifications, competences, relevance, motivation and effectiveness in providing health information. Literature data posed the question whether pharmacists and pharmacy students are prepared and motivated to use in practice the theories from such domains as psychology and sociology, and bear in mind that the psychological approach to the problem of health protection is associated with the understanding, prevention and treatment of diseases within the bio-psychological model of health and disease (Lonie and Pappan, 2010). Interestingly, in the study by Hitchings *et al.* (2013), the highest level of awareness for a safe use of paracetamol was demonstrated by pharmacists, while the lowest—by young physicians.

Medical schools during educating pharmacy students and providing post-graduate studies to pharmacists play an important role in the proper development and functioning of pharmaceutical care. The education of pharmacy students in health promotion during a 3-year programme was positively evaluated in the study by Rodis *et al.* (2015) in the United States. Letassy *et al.* (2010) demonstrated that American pharmacy students educated a group of nearly 1,900 respondents in healthy lifestyle and on the risk factors of diabetes, such as: obesity, hypertension, high level of cholesterol, or a lack of physical activity.

The results of a study by Brandys *et al.* (2009) indicate a better preparation of pharmacists aged 30–50 concerning the state of knowledge and the awareness of the problem of nicotine-addiction, compared to older pharmacists (aged over 50). The authors attribute this observation to the fact that the older generation of pharmacists is unwilling to acquire modern knowledge and lack awareness of the value of pharmaceutical care. It has already been confirmed previously in other studies, that young pharmacists (aged 30–35), compared to older ones, possess a higher level of knowledge in the area of pharmaceutical care (including the ability to present different forms of drugs), as well as a readiness to participate in educational workshops (Khan and Azhar, 2013; Amin and Chewning, 2014; Abbasinazari *et al.*, 2017). A more extensive knowledge demonstrated by the young generation of pharmacists may be explained by their awareness and positive attitude towards health promotion, as well as towards complementary courses during studies (Noureldin *et al.*, 2013; Holtzman and Sifontis, 2014; James and Bah, 2014; Justo *et al.*, 2014; Saleem *et al.*, 2015).

This study confirmed that self-assessment of the some of the domains analysed in the study: qualifications, competences, relevance, motivation and effectiveness in the area of information-health message was higher among the pharmacist working in pharmacies employing up to 3 persons, chain pharmacies, and those serving up to 100 customers daily. Pharmacy staff in Poland belong to a special occupational category, which distinguishes them from other occupational groups, due to their predispositions, knowledge and qualifications. The concept of pharmaceutical care has not yet been defined in the Polish legal system. This is due to the lack of laws regulating this concept, which causes difficulties in developing standards and principles of provision of pharmaceutical care.

European Community Pharmacy Blueprint (2012) notes the importance of pharmacists in the improvement of public health, and provides guidelines on how pharmacists employed in retailed pharmacies may contribute to the improvement of health in a society. In this strategy, it is emphasized that the provision of a wide spectrum of pharmaceutical services will contribute to a reduction in the cost of health care. It is also noted that there is a need for a greater support of pharmaceutical services provided in the area of disease-prevention and health promotion.

It is necessary to create conditions which would encourage pharmacists to improve their education in the area of pharmaceutical care. Training courses should include not only problems related with pharmacotherapy, but also with epidemiology, health promotion and especially, with communication skills in order to effectively promote health among patients.

The limitation of our study is that we did not surveyed patients or pharmacy customers and how they assess pharmacy staff in terms of health promotion and health education in pharmacies. However, we intend to do in our next study.

## CONCLUSIONS

1. The surveyed pharmacy staff provided the highest evaluations of relevance of health promotion, whereas the lowest evaluations concerned their own competences and effectiveness in health promotion, and in between these two there were evaluations of qualifications and motivation to health promotion by pharmacy staff.
2. Higher self-assessments of qualifications, competences, relevance, motivation and effectiveness in health promotion were provided by: females, the youngest pharmacy staff aged 20–29, those with a short period of employment, as well as pharmacy staff working in pharmacies employing up to 3 persons.
3. Qualification and competences in health promotion were assessed higher by pharmacy technicians and masters of pharmacy with post-graduate studies or professional specialization, or Ph.D. than by masters of pharmacy.
4. Relevance and effectiveness in health promotion were evaluated higher by pharmacy staff working in pharmacies employing up to 3 persons, being a part of the larger network, and serving on average up to more than 100 customers daily.
5. There is a need for improvement qualifications, competences, relevance, motivation and effectiveness in health promotion conducted by pharmacy staff in pharmacies in Poland.
